# Making stratigraphy in the Anthropocene: climate change impacts and economic conditions controlling the supply of sediment to Lake Geneva

**DOI:** 10.1038/s41598-019-44914-9

**Published:** 2019-07-02

**Authors:** S. N. Lane, M. Bakker, A. Costa, S. Girardclos, J.-L. Loizeau, P. Molnar, T. Silva, L. Stutenbecker, F. Schlunegger

**Affiliations:** 1 Institute of Earth Surface Dynamics, University of Lausanne, Lausanne, Switzerland; 2 Department of Civil, Environmental and Geomatic Engineering, ETH Zürich, Zürich, Switzerland; 3 Department of Earth Sciences and Institute of Environmental Science, University of Geneva, Geneva, Switzerland; 4 Department F.-A. Forel for Environmental and Aquatic Sciences, and Institute for Environmental Science, University of Geneva, Geneva, Switzerland; 5 Institute of Geological Sciences, University of Bern, Bern, Switzerland

**Keywords:** Hydrology, Limnology, Sedimentology, Geomorphology, Climate-change impacts

## Abstract

The Anthropocene has been proposed as a profound, globally synchronous rupture in the history of the Earth System with its current state fundamentally different to that of the Holocene and driven by the geological force of human activity. Here, we show how stratigraphy is being made in a lake that is heavily impacted upon by climate change and human activities. For one of the largest inner-Alpine catchments in the European Alps, we draw attention to how sedimentation rates are a product of non-stationary, reflexive, human actions. In Lake Geneva, we identify both a human-induced climate change (HCC) signature and the effects of a recent economic shock on sediment extraction upon sediment loading to and sedimentation rates in the lake. The HCC signature thus reflects the nature of climate change impacts in this basin, where sediment accumulation rates evolve with climate, but where economic conditions contribute to shifts in the supply of sediment to the lake. Following social theory, we call this glocalization because of the combined importance and inseparability of human impacts across different spatial scales. The nature of human impacts on sediment delivery to the lake mean that the influence of humans is unlikely to be captured in the long-term depositional record.

## Introduction

The Anthropocene has been identified as an abrupt transition in the Earth System^1^ driven by human activities^2^ to create a system that has no prior analogue^3^. If it is to be geologically-defined as an epoch that follows the Holocene, it must be grounded in the Geologic Time Scale^4^, and research has shown that over the last 200 years there has been a transition in depositional records. In the Holocence, records reflect the globally diachronous human impacts on landscapes and sediment systems^5^. The Anthropocene has increasingly synchronous global impacts of greater magnitude than previous epoch transitions^6,7^ and a marked acceleration of impacts from the mid-20^th^ century^8,9^. Although there is clear evidence of markers that suggest significant human forcing of the Earth System (new materials of anthropogenic origin, species extinctions and mixing, changes in the abundance of natural materials)^6,10^, the case for sedimentation rates is less clear and some have argued that sediment systems have yet to be significantly transformed^11^. This is perhaps not surprising because human impacts on sediment flux to depositional sites can be opposing. For instance, humans have dramatically increased global soil loss to the oceans due to agriculture^12^ whilst simultaneously impounding many rivers, which has reduced sediment flux substantially^13^. The balance between these opposing processes is likely to be regionally, even locally specific. There is a rich and spatially-differentiated variety of human impacts on river basins, with histories that may not be convergent^14^ in a way that would lead to a globally synchronous change in sediment balance, positive or negative, which might then be seen in the depositional record. That said, there are very few decadal-scale source-to-sink studies of what is driving the Anthropocene depositional record, and almost none in large river basins (>1000 km^2^).

In the 1990s, the word “glocalization” became a marketing buzzword, derived from Japanese business studies from the 1980s, to describe the development and marketing of global goods and services to different local markets^15^. Social theorists developed this concept to criticise a growing assumption that globalization would lead to the obliteration of local impacts on social change^15^. Glocalization has come to indicate: (a) the interaction of (social) processes at different scales, from those that were global and increasing spatial homogeneity to those that were local and increasing spatial heterogeneity; and (b) how these processes, both global and local, evolved autogenically, through self-regulation, in response to these interactions. Thus, glocalization recognizes that spatial differentiation in social processes will continue in societies despite strong globalising forces and that this differentiation will also evolve through time.

Here, we present evidence that supports the use of the notion of glocalization to frame human impacts on sedimentary systems in the Anthropocene. We illustrate this through quantifying how human-induced climate change propagates through a heavily human-impacted Alpine landscape, to change sediment delivery and sedimentation rates in Lake Geneva. We use the stratigraphic record from this lake to infer temporal and spatial patterns of sediment accumulation rates and grain size. In the upstream Rhône valley, which is the corresponding sediment source, we collect data about the provenance of the sediment, sediment fluxes, volumes of gravel extraction and perturbations of water and sediment fluxes in response to hydropower management practices. We show that a global signal related to changes in HCC-driven glacial melt is clearly visible, both in the sediment fluxes upstream of Lake Geneva and in the sediment accumulation pattern within the lake. However, grain size and sediment supply patterns have strongly been modified in response to economic and other forces in the region, with a rate of change that is unlikely to lead to centennial and longer-term, systematic changes in lake sediment stratigraphy.

## Results

### Chronology of river engineering in the Rhône basin, climate warming and glacial response

**Figure 1:**
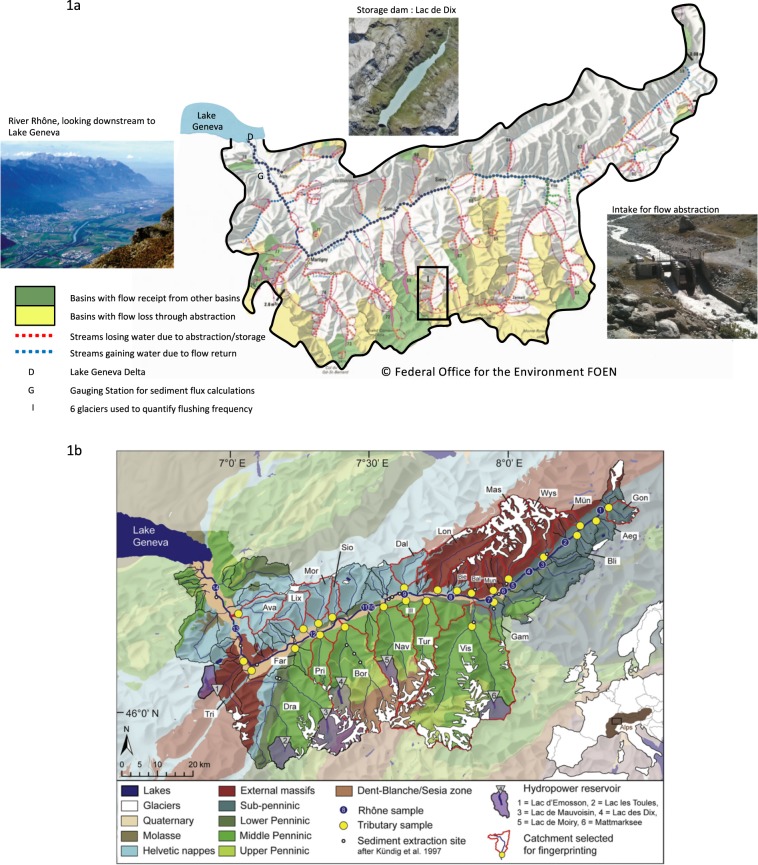
Hydropower exploitation in (**a**) and geology of the Alpine Rhône catchment (**b**). (**a**) shows examples of a storage dam, a flow abstraction intake and the embanked main Rhône. The exploitation shows the extent to which tributaries are affected by storage or flow abstraction and is based upon Margot *et al*.^48^ (**b**) illustrates the geological architecture of the Rhône basin, the sites where ^10^Be samples have been collected and analysed for basin-averaged denudation rates^21^ are also shown. These data were used to establish a sediment budget for the Rhône basin, which resulted in the notion that Penninic units are underrepresented in the total sediment flux to Lake Geneva despite highest 10Be-based denudation rates. The External massifs have supplied most of the material^21^.

Lake Geneva is the sedimentary sink of the 5’338 km^2^ Alpine Rhône basin (Fig. 1a), that ranges in altitude from 372 to 4’634 m a.s.l. It has a basin-wide average precipitation of 1’400 mm per year and unit runoff of 1’060 mm per year. Given the altitudinal range of the basin, significant amounts of precipitation fall as snow in winter and there is *c*. 730 km^3^ of accumulated ice, mainly above 2’500 m a.s.l. Geologically, the basin comprises *c*. 20% Helvetic nappes composed of calcareous sedimentary lithologies in the north-west, 25% External massifs made up of granites and gneiss in the south-west and north-east and 55% Penninic metamorphic, oceanic metasedimentary and ophiolitic rocks in the south-east and south (Fig. 1b). The Alpine Rhône river has been subject to significant human impacts, notably over the last 150 years. Two major phases (1863–1894 and 1930–1960) of engineering transformed a 103 km length of the largely anastomosing-meandering main stem into a narrow single-thread channel within dykes. The Rhône and its main tributaries have been engineered to disconnect their main channels from their floodplains and to convey delivered water and sediment through to the delta. During the 20^th^ century, and notably between 1950 and 1975, 1.2 × 10^9^ m^3^ of reservoir storage was introduced (e.g. Fig. 1) and, to increase water storage, hundreds of kilometres of inter-basin transfer tunnels were drilled. The basin is responsible for 0.44% of global hydropower potential, despite covering only 0.0041% of the global land surface. About 20% of the mean annual runoff goes into storage. The completion of the major phase of reservoir storage expansion in the 1960s was coincident with a slowing, and eventually a very slight reversal, of post Little Ice Age climate warming^16^. This was sufficient to slow retreat rates in all glaciers and lead to glacier advance by the early 1980s in many cases^17^. From the mid 1980s, there was a rapid rise in temperature to more than 1 °C warmer in the 2010s than in the 1970s^18^ and commonly attributed to HCC^19^. The focus of our work is the period from the 1950s to the present over which timescale land use in the basin is largely constant.

### Lake Geneva sedimentation rates and flux

**Figure 2:**
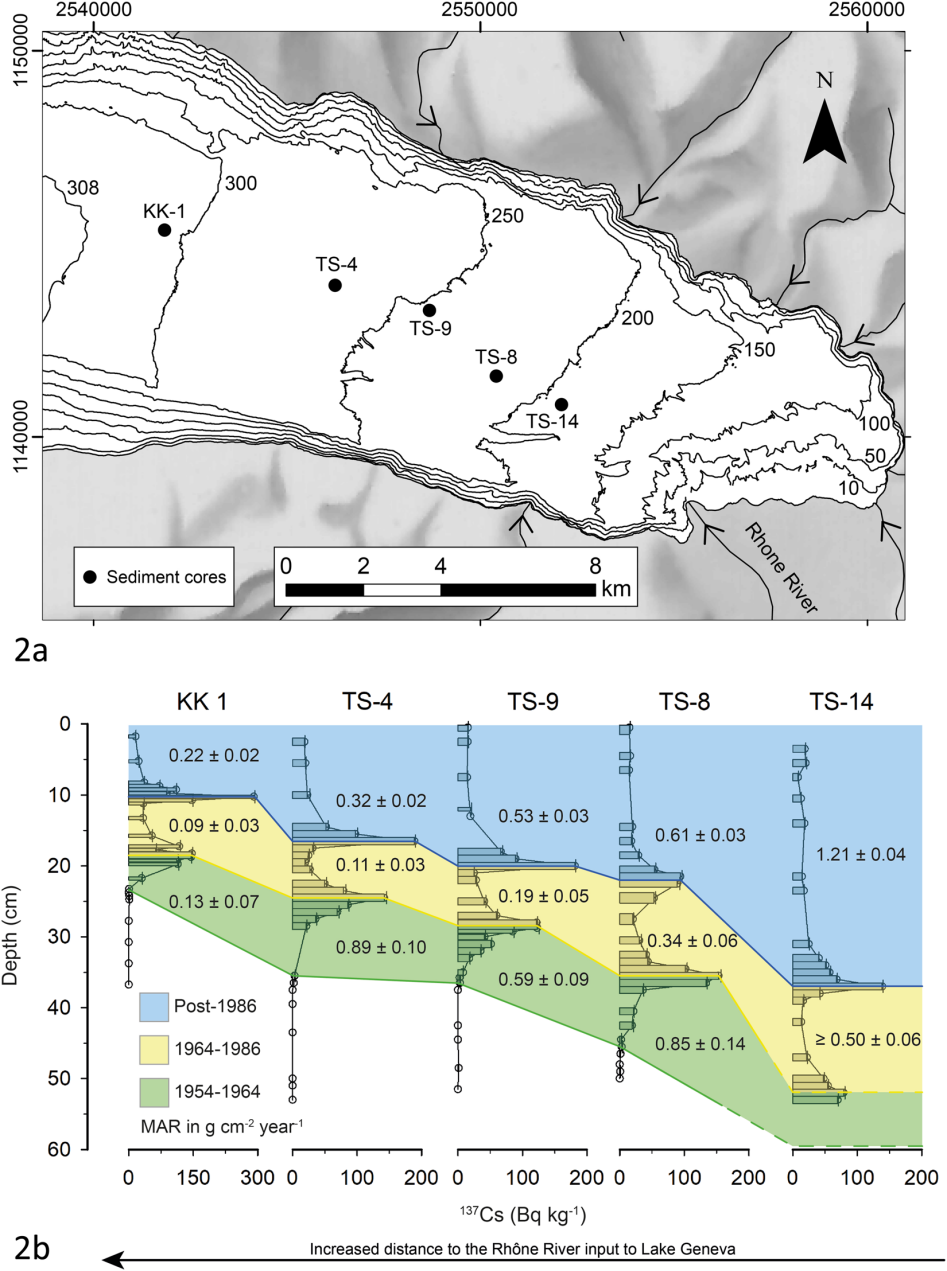
Lake Geneva showing the sites of sample cores (**a**) and the ^137^Cs-identified boundaries used to calculate the mass accumulation rates and their uncertainties shown in the Figure (see Supplementary Materials M2).

**Figure 3:**
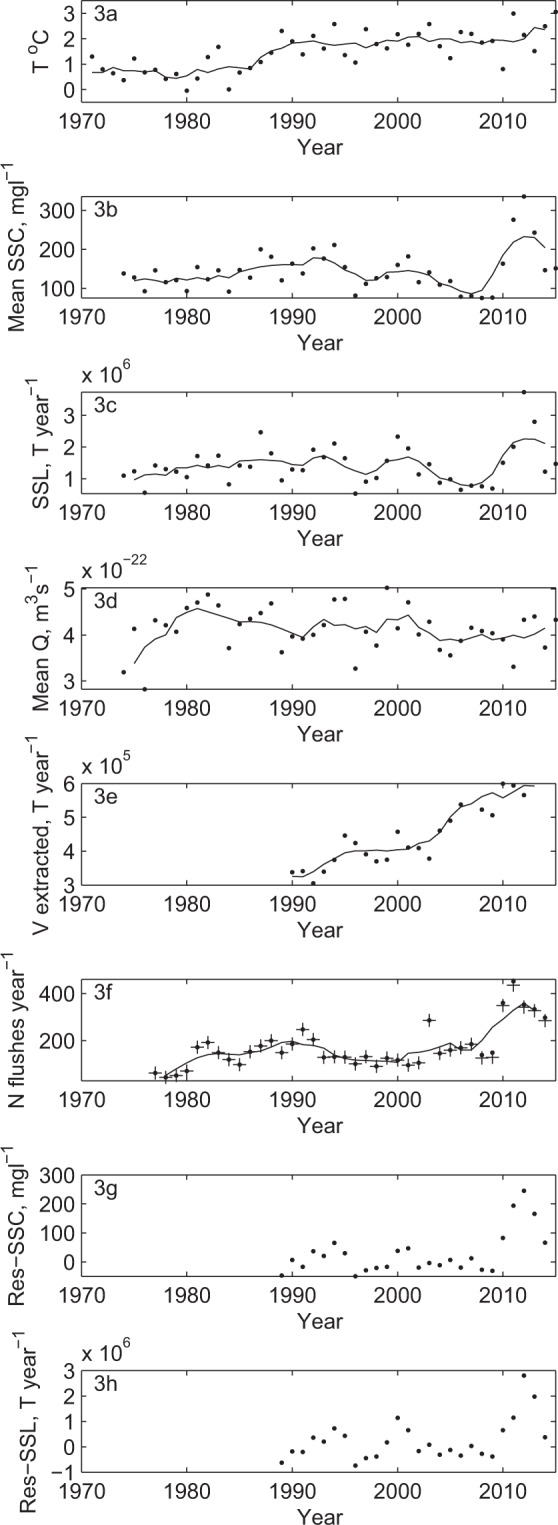
Time series of annual data for basin-averaged mean annual temperature (**a**, Supplementary Materials 1); mean suspended sediment concentration (SSC) (**b**, Supplementary Materials 3); estimated annual loading (SSL) of suspended sediment to Lake Geneva (**c**, Supplementary Materials 3), and mean annual discharge (Q) (**d**, Supplementary Materials 3) at the mouth of the Swiss Rhône (**g**), Fig. 1a); rates of sediment extraction in the Swiss Rhône basin (**e**, Supplementary Materials 4); rates of flushing of abstraction intakes (**f**, Supplementary Materials 5) in zone I on Fig. 1a; and residuals from the modelled relationship between SSC (**g**) and SSL (**h**) and sediment extraction. In (**f**), a. indicates total flushing events and a+ the minimum number of flushes given possible night time flushing (see Supplementary Materials M5).

Cores were sampled following quantification of spatial patterns of lake bed sedimentation in Lake Geneva between the late 19^th^ and early 21^st^ century^20^. Mass accumulation rates along a transect from the Rhône River mouth to the distal sub-lacustrine delta (Fig. 2a) vary spatially and temporally (Fig. 2b). Prior to 1964 and hydropower expansion, there was a clear proximal to distal reduction in mass accumulation rates in the delta. These fell significantly (t-test, *p* < 0.05) for the period 1965 to 1986 whilst the proximal to distal gradient remained. From 1987 onwards, accumulation rates have increased significantly (t-test, *p* < 0.05) for all cores to values approaching, and in one case greater than, those pre impoundment. The basin-averaged temperature record (Fig. 3a) showed a significant increase (t-test, *p* < 0.05) in mean annual temperature in the mid-1980s from 0.66 ± 0.49 °C to 1.87 ± 0.53 °C. The analysis of annual suspended sediment concentrations and sediment loads showed that the concentration of suspended sediment delivered to the lake (at G, Fig. 1a) trended upwards (Fig. 3b) from the 1970s through the 1980s to 1994 (Mann Kendall, *p* < 0.05) before declining to 2009 (Mann Kendal, p < 0.05), after which there is a rapid rise to 2014 although this latter trend is not significant (Mann Kendal, at p = 0.05). Analysis suggested a significant increase (t-test, *p* < 0.05) in the slope of the relationship between log discharge and log concentration for 1987–2014 as compared with 1974–1986. The mean annual concentrations for 1987–2014 were significantly higher than 1974–1986 (t-test, *p* < 0.05).

Annual sediment loads followed the patterns for concentration (Fig. 3c) from 1980 through to 2014. The trend to rising sediment load was significant through to 1994 (Mann Kendall, p < 0.05) and declining load was significant 1995 to 2009 (Mann Kendall, p < 0.05) after which there is a rapid rise to 2014 although this latter trend is not significant (Mann Kendal, at p = 0.05). The mean annual load for 1987–2014 was significantly higher than 1974–1986 (t-test, p < 0.05). This increased load is attributable to increased concentration as there is no significant trend in mean annual discharge from 1982 (Mann Kendal, at p = 0.05) and the relationship between log discharge and log concentration steepens from 1987 (t-test, *p* < 0.05).

Data on within-river sediment mining activities (Fig. 3e) show a significant trend (Mann Kendall, p < 0.05) in annual rates of sediment extraction for construction, combined gravel and sand, from when data begin (1989) through to 2014, and extraction rates are of a comparable magnitude to the estimated annual sediment load to Lake Geneva.

### Hydropower

Initial interpretations might follow the classic model for highly impounded river basins and attribute the reduction in sedimentation rate in the delta, from the mid-1960s, to sediment storage behind dams. However, explaining the increase in sedimentation rate (Fig. 2b) and the rising sediment loads in the 1980s and then again in the 2000s is more difficult given that no change in reservoir operation has occurred. Closer inspection of the suspended sediment concentration data showed that most of the increase was in July and August and that inter-annual variability in flux was related positively to ice melt in the basin^18^. The effects of the rapid rise in temperature and ice melt from the mid-1980s should lead to greater amounts of glacially produced fine sediment^18^. The fact that higher mean annual concentrations and loads are detected at the basin outlet (Fig. 3b,c) and have been measured in the lake stratigraphy (Fig. 2b) suggests that this climate warming signal (Fig. 3a) was able to propagate through the basin despite intense hydropower activity (Fig. 1a).

Two primary reasons were identified for the presence of an ice-melt signal despite this being a basin heavily impacted by hydropower. The first was the distribution of dams, which are primarily located in the Penninics (Fig. 1b). ^10^Be-based denudation rates were some of the highest for the Penninics (1.23 ± 0.71 mm yr^−1^)^21^, where there is also significant ice cover (Fig. 1b). It is well established that glaciers are extremely efficient erosive agents^22^, and there is growing evidence that their ability to evacuate eroded sediment is increasing due to climate driven changes in glacial hydrograph shape^23,24^; but sediment fingerprinting and mixing modelling suggested that tributaries located in the North and the East (External massifs) of the catchment were generating more of the sediment transported by the Rhône river to Lake Geneva^21^. Even though the External massifs had lower denudation rates (0.81 ± 0.39 mm yr^−1^)^21^, only the western External massif basins were impounded (Fig. 1a) such that, as climate warmed and glacial sediment supply increased, at least some of the glacial signal would be able to reach the basin outlet.

Second, many of the un-impounded Penninic and some of the External massif basins are also impacted by hydropower but with extracted water transferred laterally through tunnels to storage reservoirs in nearby valleys (Fig. 1a, yellow). Given high rates of sediment production in glaciated basins^18,25^ and to avoid the abstraction of too much sediment, the intakes have small gravel and coarse sand settling ponds (Fig. 1a). The coarse-grained material (sand fractions and coarser) is temporarily stored in these settling ponds and only washload is transferred to storage in reservoirs. When full, the sediment from the intakes is flushed down the river, which can be many times a day during peak summer ice melt. Data about flushing frequency provided by the hydropower company and the analysis of these records for 6 representative Penninic basins (I, Fig. 1a) show that flushing frequency; (1) rose markedly from the 1970s through the 1980s to 1994 (Mann Kendall, p < 0.05); (2) had no significant trend from 1995 to 2009 (Mann Kendall, at p = 0.05); after which there was a marked jump in the number of flushes per year (Fig. 3f). The average number of flushes per year was significantly lower (t-test, p < 0.05) for the period to 1986 (111 ± 17 yr^−1^) than for 1987 to 2014 (188 ± 18 yr^−1^ to 191 ± 18 yr^−1^, see Methods for explanation). The greater flushing frequency (Fig. 3f) is seen in both increased sediment delivery to Lake Geneva (Fig. 3b,c) and accumulation rates in the lake (Fig. 2b) since 1987. Flushing maintains sediment connection to downstream reaches; calculations of sediment budgets suggested that despite more than 95% flow being removed from the river at intakes and routed towards the storage reservoirs through tunnels, approximately 25% of flushed sediment went into river and floodplain storage immediately downstream. The remaining 75% of this flushed material found its way further downstream, thus maintaining significant sediment flux to the Rhône River despite the abstraction of 95% of water^26^. This sediment connection would thus allow the climate impact on ice melt and sediment export (Fig. 3f) to be propagated through the system (Fig. 3b,c) not least because greater ice melt means greater sediment load and hence an increased flushing frequency. Indeed, there was a correlation of 0.543 (Pearson’s *r*, *p* < 0.05) between flushing frequency and suspended sediment load. This relationship is also aided by the Rhône straightening and dykes (Fig. 1a), which have largely eliminated overbank flows and so floodplain sedimentation, allowing a stronger connection between delivery of sediment from upstream and the lake.

### Gravel mining

**Figure 4:**
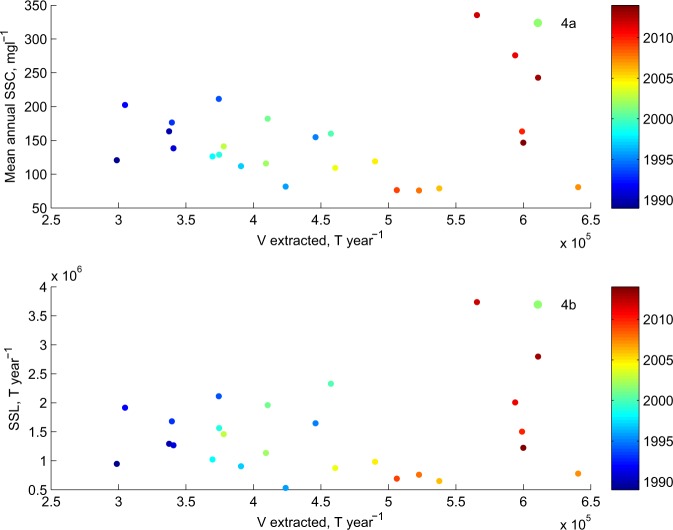
Mean annual suspended sediment concentration (**a**) and annual suspended sediment load (**b**) versus total sediment extraction. Data points are coloured by year.

Whilst the above argument suggests that a heavily hydropower-modified system was able to maintain sediment connectivity to downstream, there is one final intriguing result. There is some evidence that sediment mining in the Alpine tributaries can impact downstream sediment load even in a basin of this size. Although extracted sediment includes fractions that would not be transported in suspension, the annual rate of sediment extraction was only slightly lower in magnitude that the loading of suspended sediment to Lake Geneva. Up until the late 2000s there was a negative relationship between sediment extraction rates and both mean annual sediment concentration (Fig. 4a, Pearson’s *r* = −0.617, *p* < 0.05, 1989–2009) and suspended sediment load (Fig. 4b, Pearson’s *r* = −0.395, *p* < 0.05, 1989–2009) to Lake Geneva. We modelled this relationship for 1989-2009 using simple linear regression, used the model to predict concentrations and loads for the entire period for which we have extraction data, 1989–2014, and plotted the differences between modelled and observed extraction rates (Fig. 3g,h). The negative relationships shown in Fig. 4 for data until 2009 suggest that despite extraction data including both sand and gravel fractions, sand extraction was sufficient to be observed in downstream concentrations and loads of suspended material. However, from 2009 onwards, annual sediment concentrations and loads become independent of extraction rates (Fig. 4) and the residuals for both concentration (Fig. 3g) and load (Fig. 3h) are no longer distributed around zero but strongly positive, suggesting that extraction no longer influences them. There is a marked association with the flushing data (Fig. 3f). Sediment mining appears to be sufficient to reduce loading to Lake Geneva significantly until 2009 explaining the significant declines in load and concentration to between 1994 and 2009. However, extraction then appears to decouple from concentrations and loads and this was coincident with a significant economic downturn, and slow-down in construction rates, reported to begin from the end of 2008 by the Swiss sediment mining industry^27^.

### Discussion

We conceptualise the above trends as showing how it is the combination of global (HCC-driven) and more local processes that make the kind of depositional record that we are seeing in the Anthropocene and that is likely to be the signal seen in stratigraphical studies of such deposits in the future^28^. Whether the depositional signal of the last few decades that we address in this paper does indeed get incorporated into the permanent stratigraphic record will depend on its preservation potential. Whilst deposition rates in Lake Geneva are high close to the river mouth (cores TS-14, TS-8, TS-9, Fig. 2), this is also the zone of an active canyon^20^. Whilst the active canyon has not migrated over the last century^20^, it is likely to migrate over longer timescales. In the distal sub-lacustrine delta (cores TS-4, KK-1, Fig. 2), away from the river mouth and the active canyon, where preservation potential is highest, depositional rates were substantially lower^20^. In the zone of highest preservation potential, we identify a signal that for the moment can only be identified using micro-techniques (e.g. microscopes). However, this signal is also non-stationary, with a period of lower sedimentation until the mid-1980s, followed by a rise from the mid-1980s. A primary reason for this non-stationarity is that human impacts on sediment flux to the delta, whether related to global climate change (here increasing ice melt and sediment supply) or water and sediment management (here related to hydropower, river management and sediment extraction) are potentially opposing and evolve through time. On the one hand, evidence suggests that climate warming is driving glacial melt-driven sediment export^18,29^. This can still be seen downstream of dams and intakes because the hydropower system operation is strongly coupled with climate (Fig. 3f); as climate warming produces more ice melt, it also produces more sediment, and hence a greater need to flush intakes. On the other hand, until the economic shock of 2008, sediment extraction (Fig. 3e) seemed to grow with sediment supply (Fig. 3f) and this was sufficient to cause lower concentrations and loads at the Lake during the 1990s and early 2000s (Fig. 4). Sediment extraction has partly desensitised sediment delivery to the Lake from upstream climate warming. Yet, these relationships are also non-stationary. Here, an external economic shock slowed the growth in sediment extraction rates from 2009 onwards just as there was growing sediment export from glaciated parts of the basin. The result was a very rapid rise in sediment concentration and loading to the highest values on record (Fig. 3b,c). Given the close relationship between ice melt and glacial sediment export^18,29^, it is likely that as the ice volume in the basin declines and a state of “peak water” is passed, here predicted for the 2030s to 2050s^30^, so annual sediment delivery will reduce. This process may also be reinforced by the progressive reduction in sediment availability in deglaciated zones as the landscape passes through its paraglacial phase^31^. It is this combination of HCC with human impacts within the basin that can: (a) result in opposing tendencies; (b) make sediment delivery prone to external shocks; and (c) involve co-evolution of drivers (e.g. climate, human response) within the system.

Our data show that the exact nature of human impacts on the processes that influence sediment depositional environments will have substantial variability, within and between basins, as well as through time^32,33^. Although evidence of a “golden spike”, that is a sudden stratigraphic break, is clear for a number of markers (new materials of anthropogenic origin, species extinctions and mixing, changes in the abundance of certain materials)^6,10^, indeed we use such a marker ourselves to estimate mass accumulation rates in Lake Geneva in this paper, the case for sedimentation is much less clear. As others have argued, whilst sediment systems are being significantly transformed by human activities^12^, this is unlikely to be globally coherent and synchronous^6,11^. It is perhaps remarkable that short-term sedimentation rates respond so rapidly to global forcing and the speed of response means that the human signal in sediment delivery to Lake Geneva is unlikely to be stationary for sufficient time (decades and longer) to be seen in significant changes in sedimentary layers. The richness and complexity of human impacts, and the reflexive capacity of humans to respond to those impacts, is at once broadening the spatial-scales (impacts of global temperature rise; large-scale hydropower investment; global economic shocks) but collapsing the time-scales over which global change becomes recorded; and leading to high non-stationarity in the depositional record. We argue that it is these changing space and time-scales that distinguish the Earth System in the Anthropocene as compared with previous epochs. It will likely be very hard to define an Anthropocene stratigraphic unit (e.g., a member or a formation) based on sediment stratigraphy alone.

Our example is not the only one to support the reflexivity of human impacts. For instance, in one of the most impounded countries in the world, the USA, awareness of the environmental impacts of dams, including upstream sediment accumulation^34^ and downstream sediment starvation^35^, is leading to both flood releases to re-establish sediment flux and dam removal. Such changes are likely to re-establish the impacts of human activity on soil erosion, downstream sediment deposition and reverse current rates of delta sinking^36^. We propose the concept of glocalization as the means of conceptualizing the opposing, co-evolving and non-stationary signatures of human impacts on sedimentation rates. Glocalization occurs in four ways: (1) global processes (e.g. rises in greenhouse gas concentrations and global increase in temperatures paired with larger ice melt; economic downturn) have regional and local scale manifestations (here in terms of Alpine climate change; changes in demands for extracted sediment); (2) the river basin itself conditions the impact of these processes (e.g. where sediment can be eroded, whether sediment compartments are connected, and whether sediment can be transported^37,38^); (3) humans modify these processes (e.g. dams, intakes, flushing, storing and mining). In addition, and most interestingly (4) these modifications are reflexive, whether implicitly, such as built into infrastructure (e.g. increases in intake flushing frequency forced by changing climate) or explicitly, here as the downturn in the construction industry in the late 2000s slowed the growth in sediment extraction rates, so reducing mining impacts on sediment concentrations and loading to Lake Geneva, and leading to very high suspended sediment concentrations and loading to the Lake. As a framing of the Anthropocene, the term glocalization gains its explanatory power not simply because it recognises that the stratigraphic record is going to be a product of both global processes and their conditioning by local effects; but also because it emphasises that interactions between different scales in a human-impacted world are continual and reflective; an additional kind of autogenic response^39,40^ to the forcing of erosion and sediment transport through river basins that arises from physical, chemical or biological processes. We do not deny the dramatic impacts that humans are having on sediment flux over large areas and short timescales. However, we argue that it is highly unlikely that such impacts will last for long enough to be detectable in the sedimentary record. We must therefore avoid naïvely searching for globally synchronous human impacts upon rates of sediment deposition.

## Methods

### Climate data

Climate data were provided by MeteoSuisse on a 2 × 2 km grid with a daily resolution. These were averaged to the basin scale.

### Determination of delta sedimentation rates and sediment composition

The coring sites in Lake Geneva were selected to form a longitudinal profile highlighting the spatial evolution of mass accumulation rates (MARs) with distance from the Rhône River delta. Sample locations were based upon a study that quantified spatial patterns of lake bed sedimentation in Lake Geneva between the late 19^th^ and early 21^st^ century^20^ to identfify the the mean ‘background’ sedimentation in upper Lake Geneva^41^. Core locations were not influenced by the internally- or externally-triggered mass-transport deposits and turbidites that occur in the active Rhone canyon^42^ over the decadal timescale that we report. Four sediment cores were collected in 2014 and one core KK1 in 2010^43^ using a gravity corer (Uwitec, Austria) with transparent 59.5-mm-diameter PVC tubes.

After collection, sediment cores were stored at 4 °C until they were split longitudinally in two halves using a Geotek core splitter (Geotek Ldt, UK). For each core, one half was wrapped in plastic and stored in a cold room as an archive, while the other half was sub-sampled at 1-cm resolution. Turbidites, which were identified in the sediment record based on changes in grain size and colour, were sampled separately.

Water content was determined in each sample by wet and dry weight difference. Porosity was calculated from water content, assuming that the samples were water-saturated, and using a particle density of 2.6 g cm^−3^. The mass of particles per square centimeter was calculated from porosity and particle density in each sample and then cumulated to express the vertical depth in sediment cores as cumulative mass depth, eliminating the effect of sediment compaction^44^.

Sediment cores were dated through gamma-ray spectroscopy (^137^Cs isotope activity). Samples were measured individually in High Purity Germanium (HPGe) well detectors (Ortec GWL series, USA). Three time-horizons were determined from ^137^Cs activity, from bottom to top core: 1954 as the first occurrence related to the fallout from the atmospheric nuclear tests, 1964 as the first peak linked to the maximum fallouts from these tests and 1986 as second peak due to the Chernobyl accident^45^. MARs were then calculated by the total mass of sediment deposited between each time-horizons divided by the time span between them.

### Determination of mean annual suspended sediment concentrations and sediment loads

Suspended sediment concentration (SSC) is sampled twice weekly by the Swiss Federal Office of the Environment at Port du Scex (G1, Fig. 1a, main paper). These data were used to determine annual mean SSCs and suspended sediment loads (SSLs), and hence delivery rates, to Lake Geneva. Given diurnal and seasonal scales of discharge variation and hence suspended sediment concentration, annual mean SSCs needed to be weighted through a rating curve, also needed for the determination of SSLs. Continuous hourly mean discharges (Q) for the same station were matched with corresponding SSCs. Log-log Q-SSC rating curves were constructed for each year of measurement using least squares regression. As such curves are fit in log-log space, their use to estimates SSCs from continuous discharge measurements are biased and so we applied Monte Carlo simulation in log-log space to estimate 1000 SSCs for each hourly measure of Q, transform these into linear space and then take the average^46^. Tests suggest that 1000 simulations was more than enough to obtain stable estimates of SSC. Hourly SSC values were averaged across the year to obtain annual mean SSC values. They were also combined with hourly Qs to obtain hourly loads. These were summed to obtain annual loads.

### Within-river sediment mining

Data on all sediment mining from within the basin, the main Rhône river and tributaries, were obtained from the Canton of Valais’s Department of Transport, Infrastructure and Environment. Following cantonal law, all extraction of sediment from rivers that exceeds 10’000 m^3^ per year has to be authorised and recorded. These data were available from 1989 to 2014.

### Basin scale denudation rates, sediment finger printing and mixing modelling

Data on basin scale denudation rates were obtained using concentrations of *in-situ* ^10^Be measured on river born quartz^21^. Samples were taken from tributary streams of the Rhône river, and along the Rhône itself. Sediment fingerprinting was established through framework petrography, heavy mineral concentrations and bulk geochemistry of detrital sand, collected from tributary streams and along the Rhône river. The relative contribution of the various tributary basins on the bulk sediment budget of the Rhône river was accomplished through the Optquest algorithm, that allows the identification of the percentage contributions of end-member sources. This approach has been considered to best reproduce the observed in-stream sediment composition by minimizing the difference between simulated and observed composition^21^.

### Flushing frequency

By working with the hydropower company Grande Dixence SA, we were able to secure 15 minute resolution discharge data from 1968 to 2014 for six basins with glaciers in the Borgne river, a left bank tributary of the Rhône River (I, Fig. 1a, main paper). The characteristics of these basins are given in Table 1.

**Table 1:** Characteristics of basins used to determine flushing frequency^49^.

Parameter	Bertol	Douves Blanche	Haut Glacier d’Arolla	Vuibé	Pièce	Tsijiore Nouve
Basin size upstream of gauge (km^2^)	2.51	1.01	12.65	2.27	2.79	4.77
% glaciated in 1973	21.9	21.8	45.9	90.0	52.8	67.0
% glaciated in 2009	13.6	6.9	27.2	NA	45.1	57.1
1973 snout altitude	2840	2980	2560	2700	2630	2223
1973 glacier mean altitude	3080	3220	2960	3600	2911	3300
1973 glacier maximum altitude	3300	3360	3480	3795	3695	3770
Aspect	SW	SW	N	NE	N	NNE

All of these basins are exploited for hydropower, with an intake in each, either a single intake or a double pass intake, the first designed to settle out gravel and the second designed to settle out coarse sand, before abstracted water is transferred in tunnels to a storage lake, Lac de Dix. For regulatory reasons, each intake has to record precisely the volume of water (in m^3^) that is abstracted. Thus, each has a broad-crested weir that has been calibrated to provide discharge data. However, once an intake is full of sediment, it has to be flushed, and this can be many times per day for the bigger basins with large glaciers (notably the Haut Glacier d’Arolla and Tsijiore Nouve, Table 1). When the intake is flushed, the discharge falls rapidly and such drops can be seen clearly in discharge records (Figure M5.1). In almost all cases, flushing is related to the intake being full or nearly full with sediment and hence by counting the number of flushes per year, we can get an index of the level of activity in the upstream basins. There is an exception to this, for the Haut Glacier d’Arolla and Tsijiore Nouve, the operator introduced a different intake management system from 2008. For safety reasons, from this year, rather than waiting for when the intake was full, the intake was flushed at 23.30 each evening provided the intake was at least half full. This will increase the number of flushes recorded. Hence, from 2008 onwards, we calculate a range of total flush numbers, a maximum assuming that Haut Glacier d’Arolla and Tsijiore Nouve were full at evening flushing, and a minimum assuming they were only half full at night time flushing. The method used to identify flushes for the Haut Glacier d’Arolla, detailed in (29) is used for all other intakes except Tsijiore Nouve, where the method is detailed in (26). The data were used to determine total annual flushing rates for the six basins as an index of glacial sediment production. Work^29,47^ has shown that flushing is primarily driven by glacial melt because of the importance of the latter for connecting subglacially eroded sediment to the basin outlet.

## Supplementary information

Dataset 1

## Data Availability

The data used in this project will be made available through the Alpine geo-portal ebibalpin.unil.ch and are also available in Supplementary Material.
